# Environmental Toxins and Oxidative Stress: The Link to Cardiovascular Diseases

**DOI:** 10.3390/antiox14050604

**Published:** 2025-05-17

**Authors:** Rasheed O. Sule, Gabriela Del Toro Rivera, Tanishq Vaidya, Emily Gartrell, Aldrin V. Gomes

**Affiliations:** 1 Department of Neurobiology, Physiology, and Behavior, University of California, Davis, CA 95616, USA; rosule@formerstudents.ucdavis.edu (R.O.S.); gcrivera@ucdavis.edu (G.D.T.R.);; 2 Center for Mitochondrial and Epigenomic Medicine, Children’s Hospital of Philadelphia, Philadelphia, PA 19104, USA; 3 Department of Physiology and Membrane Biology, University of California, Davis, CA 95616, USA

**Keywords:** air pollution, inhaled particulate matter, lead, mercury, microplastics, mold, oxidative stress, ER stress, mitochondrial dysfunction, cardiovascular diseases

## Abstract

Cardiovascular diseases (CVDs) remain a leading global health concern, responsible for substantial morbidity and mortality. In recent years, as our understanding of the multifaceted nature of CVDs has increased, it has become increasingly evident that traditional risk factors alone do not account for the entirety of cardiovascular morbidity and mortality. Environmental toxins, a heterogeneous group of substances ubiquitous in our surroundings, have now entered the spotlight as offenders in the development and progression of CVDs. Environmental toxins include heavy metals, air pollutants, pesticides, and endocrine-disrupting chemicals, among others. Upon exposure, they can elicit oxidative stress, a condition characterized by an imbalance between the production of reactive oxygen species (ROS) and the body’s ability to detoxify and repair the resulting damage. Oxidative stress triggers a cascade of events, including inflammation, endothelial dysfunction, lipid peroxidation, and vascular remodeling, which can contribute to the development of atherosclerosis, hypertension, and other cardiovascular pathologies. This article delves into the molecular mechanisms underpinning oxidative stress-mediated cardiovascular damage induced by environmental toxins, emphasizing the role of specific toxins in this process. Further research is necessary to understand how individual susceptibility and genotype influence the impact of environmental toxins on oxidative stress and the risk of CVD.

## 1. Introduction

In recent decades, the growth of large-scale industrial processing and increasing reliance on fossil fuels have contributed to a boom in the ambient levels of known environmental toxins [1]. These toxins and their byproducts are defined as poisonous substances that adversely impact human well-being and are now released into the environment at almost 220 billion tons per annum [2]. Levels of toxins, such as particulate matter in the air and toxic metals, are continually rising and are now considered key contributors to human mortality. For example, pollution-associated diseases accounted for nearly 16% of all deaths worldwide in 2015 [3]. Research into the mechanism of exposure to these hazardous agents has shown that exposure predominantly occurs via inhalation, ingestion, and absorption through the skin [4]. Environmental toxins have an impact not only on physical health but are also deeply intertwined with human social issues. Research into the distribution of environmental toxins from around the world has shown that low- and middle-income communities are disproportionately exposed to environmental pollution [1], further contributing to lowered health equity. This is of dire concern as demographic shifts predict that by 2050, nearly 50% of the global population will reside in crowded urban locations, potentially exacerbating this crisis [5]. Within the multitude of harmful effects caused by these toxins, several epidemiological studies have linked environmental toxin exposure with an increased risk of cardiovascular disease (CVD) [6].

Since CVD remains the number one cause of death amongst developed nations, substantial work has been undertaken to elucidate the causal mechanisms driving this cardiotoxicity [7]. This research has implicated environmental toxins with a variety of harmful pathologies, ranging from increased oxidative stress, increased systemic inflammation, increased risk of atherosclerosis, and impaired cardiomyocyte contractility [6,8]. Although previous studies have elucidated some key mechanisms that can contribute to CVD, a comprehensive understanding, particularly relating to the detailed molecular changes involved and the interactions of pollutants found in the environment, is lacking [9,10].

Despite significant improvements in addressing established cardiovascular risks, environmental toxins remain underrecognized yet are substantial contributors to the global CVD burden. As such, understanding this multifaceted link between CVD and environmental toxins is of utmost importance, as only through a comprehensive understanding can effective, comprehensive public health solutions be devised to minimize toxin exposure. Knowledge about how environmental toxins drive oxidative stress and contribute to CVD risk is not only a scientific pursuit but also a pressing public health imperative.

The purpose of this review is to synthesize current evidence on the critical relationship between environmental toxins and CVDs, with a specific focus on oxidative stress as a central mechanistic pathway.

## 2. Methodology

PubMed and Web of Science were systematically searched to identify applicable studies. Search terms included combinations of “environmental toxins”, “heavy metals”, “air pollutants”, “particulate matter (PM2.5/PM10)”, “tobacco smoke”, “radiation”, “pesticides”, “endocrine-disrupting chemicals (EDCs)”, “bisphenol A (BPA)”, “mold”, “pharmaceuticals”, “personal care products”, “dioxin”, “asbestos”, “arsenic”, “cadmium”, “lead”, and “mercury”. For cardiovascular outcomes, the following search terms were used in combination with the other search terms above: “cardiovascular diseases”, “atherosclerosis”, “hypertension”, “myocardial infarction”, and “endothelial dysfunction”. To determine the mechanistic pathways involved, the search terms “oxidative stress”, “reactive oxygen species (ROS)”, “lipid peroxidation”, “inflammation”, and “antioxidant” were used in combination with the other terms listed above. Boolean operators (AND/OR) linked these terms, and MeSH headings were applied in PubMed [1]. Studies published from inception to January 2025 were included to find former and recent advances in environmental toxins and cardiovascular disease.

The inclusion criteria included peer-reviewed articles that explored the role of environmental toxins in CVDs via oxidative stress pathways and human and animal studies, both in vitro, in vivo, and observational studies, as well as studies reporting quantitative associations between toxin exposure and cardiovascular outcomes (e.g., biomarker levels). The exclusion criteria included studies focusing solely on traditional CVD risk factors (e.g., hypertension and diabetes) without environmental toxin linkages, research lacking a clear analysis of oxidative stress mechanisms, and non-English articles, editorials, and conference abstracts. To ensure reproducibility and rigor, all systematic reviews and meta-analyses from 2022 to 2024 were hand-searched to identify overlooked studies. This methodology aligns with PRISMA (Preferred Reporting Items for Systematic Reviews and Meta-Analyses) guidelines [2].

## 3. Common Environmental Toxins

The most common environmental toxins include the following:

**Air Pollutants:** These include particulate matter (PM2.5 and PM10), ground-level ozone (smog), nitrogen dioxide (NO_2_), sulfur dioxide (SO_2_), carbon monoxide (CO), and volatile organic compounds. Sources of air pollutants include industrial emissions, vehicle exhaust, and combustion processes.

**Heavy Metals:** Lead is found in older paints, pipes, and contaminated soil. Mercury is present in certain fish species due to water contamination. Arsenic is found in some drinking water and foods.

**Pesticides**: Chemicals used in agriculture and pest control, such as organophosphates and chlorpyrifos, can find their way into the food supply.

**Endocrine-Disrupting Chemicals (EDCs)**: These chemicals can interfere with hormone function and include substances like bisphenol A (BPA) found in some plastics and phthalates found in personal care products.

**Polychlorinated Biphenyls (PCBs)**: PCBs were historically used in electrical equipment but are now banned mainly due to health concerns. However, they persist in the environment.

**Dioxins**: Dioxins are released into the environment through industrial processes like waste incineration and can accumulate in the food chain.

**Asbestos**: Asbestos is a fibrous mineral that, when disturbed, can release harmful airborne fibers. In the past, it was often used for building materials.

**Radon**: Radon is a naturally present radioactive gas that can leak into homes from beneath the ground.

**Tobacco Smoke**: Tobacco smoke contains numerous toxins, including nicotine, carbon monoxide, and various carcinogens.

**Mold and Indoor Air Contaminants**: Mold spores and indoor allergens can pose health risks, especially to individuals with allergies or respiratory conditions.

**Pharmaceuticals and Personal Care Products**: Residues from medications and personal care products can enter water supplies and affect aquatic ecosystems.

**Radiation**: Exposure to ionizing radiation from sources like nuclear power plants, medical procedures, and certain industrial activities can be harmful.

**Chemical Waste and Spills**: Accidental spills of hazardous chemicals, like oil spills or chemical plant accidents, can have significant environmental and health impacts.

**Microplastics**: Microplastics are tiny plastic particles found in the environment, including in water and food sources.

Importantly, all of these environmental toxins cause oxidative stress (Table 1).

## 4. Oxidative and Antioxidative Stress

**Figure 1:**
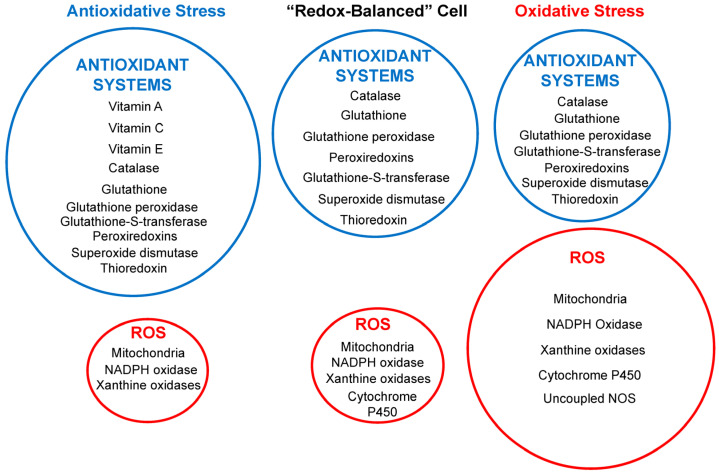
Schematic diagram illustrating the relative contributions between the generation of reactive oxygen species (ROS), shown in the red circles, and the presence of antioxidant systems (in blue circles) within cells in three different potential intracellular environments. The circle sizes correspond to the antioxidant system’s protective power relative to ROS generation levels. NOS, nitric oxide synthase.

The balance between the production of reactive oxygen species (ROS) and the presence of antioxidant systems within cells dictates the cell’s antioxidant status (Figure 1). Three related but distinct concepts in the field of redox biology are oxidative stress, antioxidative stress, and redox-balanced cells [115]. Under normal conditions, low ROS levels act as signaling molecules supporting cardiovascular function or are removed by the cellular antioxidant systems, such as superoxide dismutase (SOD) and glutathione (GSH), resulting in a redox-balanced cell. However, environmental toxins such as air pollutants and heavy metals can disrupt this equilibrium, resulting in excess ROS that overwhelm antioxidant systems, referred to as antioxidative stress. Environmental toxins such as PM cause oxidative stress, which occurs when there is an excess production of ROS that the intrinsic antioxidant systems in cells cannot adequately neutralize.

A redox-balanced cell can adjust its antioxidant capacity according to the changing redox environment and can cope with mild or transient oxidative stress without compromising its viability or performance [115]. However, an often overlooked antioxidant status is antioxidative stress, whereby under conditions of overconsumption of synthetic antioxidants, the levels of ROS can become lower than required to support some cardiovascular function, leading to harmful effects [99,116,117]. Antioxidative stress can be achieved by upregulating the endogenous antioxidant systems, such as glutathione, superoxide dismutase, catalase, or peroxiredoxin, or by supplementing with exogenous antioxidants, such as antioxidants, such as vitamin C, vitamin E, or polyphenols. It may be the cause of some of the adverse effects of antioxidant therapy discussed in the clinical implications.

**Figure 2:**
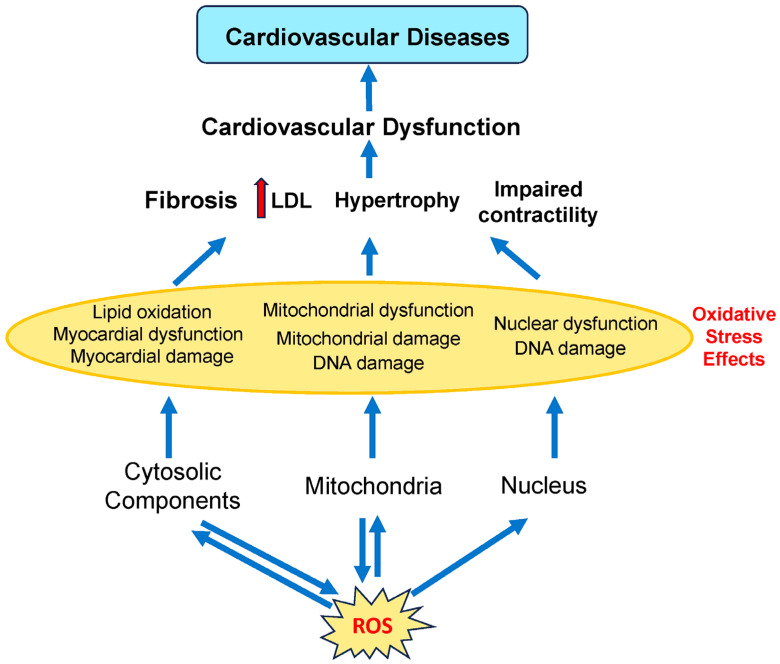
Involvement of different subcellular components of cardiovascular cells in cardiovascular diseases. ROS is produced by the mitochondria and other organelles found in the cytoplasm (such as the endoplasmic reticulum and peroxisomes). ROS produced by the mitochondria and other organelles act on both mitochondrial and cytosolic components. Cardiovascular diseases are caused by mitochondrial, myocardial, and nuclear dysfunction. LDL, low-density lipoprotein. Red arrows indicate increased levels of that component. The dark blue arrows indicate the direction of signaling.

Enzymes present in several organelles can produce ROS, including xanthine oxidases (XO) in peroxisomes, NADPH oxidase (NOX) in the endoplasmic reticulum and plasma membranes, cytochrome P450 (CYP) in the endoplasmic reticulum, XO in the peroxisomes, and the electron transport chain (ETC) in mitochondria (Figure 2). Intracellular ROS dynamically interacts with mitochondria, cytoplasm, and the nucleus, creating a bidirectional regulatory network.

High ROS levels damage lipids, proteins, and DNA, driving endothelial dysfunction, inflammation, and atherosclerosis. Lipid peroxidation byproducts, such as 4-HNE (4-hydroxynonenal) and MDA (malondialdehyde), disrupt cell membranes, promoting cardiomyocyte death and compensatory fibrosis [118]. Environmental exposures amplify these effects by directly increasing ROS generation and impairing antioxidant capacity. While the mitochondria are a major source of ROS from complexes I and III, ROS also damages mitochondrial proteins, causing mitochondrial damage and dysfunction. ROS overproduction from dysfunctional mitochondria induces oxidative damage to proteins and DNA, impairing ATP synthesis and contractility [119]. Cytoplasmic ROS, such as from NOX, activate signaling pathways but also induce oxidative damage to proteins and lipids. Excessive cytosolic ROS can cause DNA damage, resulting in nuclear dysfunction.

Though mitochondria, cytoplasm, and the nucleus exhibit compartment-specific mechanisms for redox homeostasis, they engage in dynamic interorganellar cross-talk via ROS-mediated signaling. ROS from the mitochondria can go to the cytoplasm. Similarly, ROS produced in the cytoplasm can travel back to mitochondria and affect their energy production. Cells maintain healthy ROS levels by having specific control systems in each compartment, all working together like a coordinated team.

To discuss all 14 environmental toxin groups in significant depth would result in a review of over 20,000 words, which would be too extensive for a single article. A more focused review of five toxins (air pollutants, heavy metals, PPCPs, mycotoxins, and microplastics) was selected based on their widespread exposure, diverse mechanisms of inducing oxidative stress, and the strongest mechanistic evidence for ROS production specifically linked to cardiovascular outcomes. Other toxins, while important, either have more specialized effects or overlap with these primary categories in terms of oxidative stress mechanisms. For example, while radiation and radon cause oxidative stress through ionization, these effects are more related to DNA damage and cancer rather than cardiovascular diseases. While pesticides induce oxidative stress, their mechanisms are somewhat similar to those of heavy metals and mycotoxins, involving mitochondrial disruption and antioxidant suppression.

## 5. Air Pollutants—Particulate Matter (PM)

**Figure 3:**
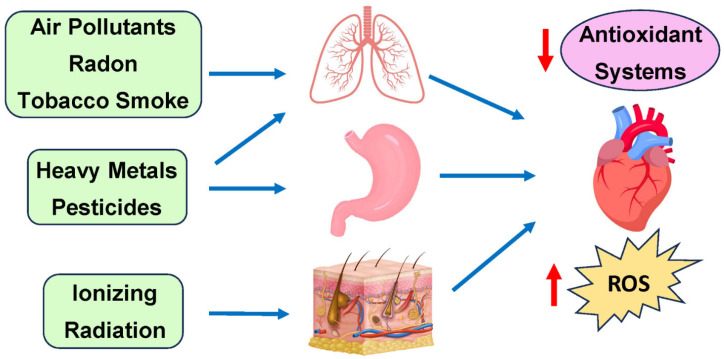
Primary exposure routes of environmental toxins and their cardiovascular effects.

A major environmental toxin implicated in increasing the risk for CVD is air pollution based on ambient PM. Specifically, ambient PM is defined as the minute solid particles or liquid droplets suspended in the air that can be produced because of human or natural activities [68]. In most cases, ambient PM consists of a heterogeneous mixture of particles of multiple sizes and chemical compositions, including compounds such as sulfur dioxide, nitrogen dioxide, and volatile organic compounds. Due to their varied nature, PM is classified in terms of its particulate sizes. PM10 refers to particulate matter with an aerodynamic diameter (AD) ranging from 2.5 to 10 μm and typically includes coarse particles originating from various sources such as road and agricultural dust, construction, and mining operations [69]. PM2.5, which includes fine particles with an AD less than 2.5 μM, is predominantly from power plant fuel combustion and mobile brake emissions. The last category of PM is ultrafine particles (UFPs), which include particles with diameters less than 0.1 μM. These mainly come from tailpipe emissions from mobile sources. Primary exposure to PM occurs via the respiratory system and eye conjunctiva, with some contribution to the gastrointestinal tract. Beyond direct air contact, certain pollutants can enter the human body through food (Figure 3) [70]. Presently, it is estimated that around 4.2 million deaths can be attributed to ambient air pollution and 2.9 million to household air pollution [3,71]. Furthermore, rapid urbanization has played a role in exposing over 90% of the world’s population to air pollution levels that exceed the World Health Organization (WHO) air quality guidelines [72].

Environmental toxins initially affect different tissues: ionizing radiation impacts the skin (low/moderate doses) or the heart (high doses); air pollutants, radon, and tobacco smoke target the lungs; while heavy metals and pesticides enter through digestion. Systemic effects from these exposures indirectly drive cardiovascular diseases, primarily through increased ROS production and decreased antioxidant defenses in heart tissue.

In terms of cardiovascular pathologies specifically, PM has been associated with conditions such as increased risks of myocardial infarction (MI), stroke, arrhythmia, and heart failure exacerbation [73]. Numerous epidemiological sources have attributed PM2.5 to the key group responsible for cardiovascular incidents. For example, several epidemiological studies have implicated PM2.5 in cases of CVDs, such as coronary artery disease [74,75,76], acute MI [76,77,78], cardiac arrhythmias [79,80], and hypertension [81]. This vast amount of evidence has led the American Heart Association (AHA) to claim a causal link between PM2.5 exposure and cardiovascular morbidity and mortality [73,82]. Furthermore, both short- and long-term exposure to PM2.5 has been associated with increased rates of cardiovascular damage [73].

**Figure 4:**
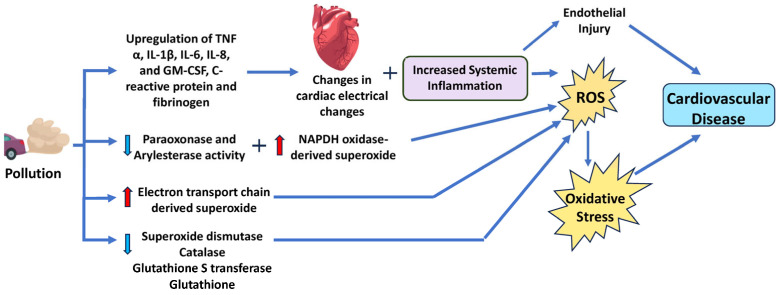
Schematic diagram of the mechanism and signaling pathways by which particulate matter increases cardiovascular disease risk. Particulate matter induces ROS and reduces antioxidant systems, resulting in oxidative stress. IL, interleukin; TNF-α, tumor necrosis factor-α; GM-CSF, granulocyte-macrophage colony-stimulating factor. Red arrows indicate increased levels of that component, while light blue arrows show decreased levels of that component. The dark blue arrows indicate the direction of signaling.

While the exact mechanisms through which particulate matter contributes to CVD are not fully understood, research suggests that much of their effect stems from their ability to generate ROS [83,84,85]. Several studies have demonstrated PM’s ability to increase markers of cellular oxidative stress [74,86,87]. This effect is also seen in a wide range of cells, including nasal, airway, and lung epithelial cells [88], macrophages [89], and cardiomyocytes [90]. For example, PM exposure in alveolar macrophages has been associated with the transcriptional upregulation of inflammatory cytokines, including TNF-α, IL-1β, IL-6, IL-8, and GM-CSF [91,92]. These markers indicate increased systemic inflammation, which can lead to subclinical responses such as increased vasoconstriction and cardiac electrical changes (Figure 4). These subclinical pathologies then contribute to an increased risk of acute ischemic or thrombotic events, atherosclerosis, hypertension, and chronic heart failure [93]. Altering the balance between pro- and anti-inflammatory cytokines, such as TNF-α and IL-6, can affect the recruitment and activation of immune cells, the secretion of chemokines, and the regulation of apoptosis and tissue repair. Another possible mechanism is via the nicotinamide adenine dinucleotide phosphate (NADPH) oxidase (NOX), which is a key contributor to cellular ROS and atherosclerosis (Figure 4). Furthermore, a study by Kampfrath et al. demonstrated that chronic exposure to PM2.5 increased NADPH oxidase-derived superoxide in monocytes and aortic tissue [94]. Other sources of PM-induced ROS include increased leaking of electrons from the mitochondrial ETC, and reductions in catalase (CAT), glutathione S transferase (GST), and SOD activity, and glutathione (GSH) levels [11,12].

Lin et al. also demonstrated that traveling from Los Angeles (a less polluted area) to Beijing (a more polluted area) lowered the antioxidative activities of paraoxonase and aryl esterase as well as increased proinflammatory C-reactive protein and fibrinogen (Figure 2) [95]. Thus, there is strong support to suggest that the cardiotoxic activity of PM, especially PM2.5, stems from its ability to increase systemic inflammation. Additionally, Su et al. exposed C57BL/6 mice to PM2.5 or filtered air for 8 or 16 weeks and found that cardiac hypertrophy developed in PM2.5-exposed mice might be regulated by the PI3K/Akt/FoxO1 pathway [13]. In cardiomyocytes, the activation of the PI3K/Akt pathway led to hypertrophic growth and net protein accumulation due to growth factors secreted [96]. Therefore, PI3K/Akt can trigger its downstream protein FoxO, regulate cardiac cell catabolism, and inhibit cell growth by specifically targeting hypertrophy signal molecules [96]. Previous studies have indicated that FoxO plays a role in the development of different kinds of heart diseases [97]. This implies that the PI3K/Akt/FoxO1 pathway might be responsible for cardiac hypertrophy due to exposure to particulate matter, thereby increasing cardiovascular risk. PI3K is a key enzyme that mediates cellular responses to growth factors, hormones, and cytokines and can be activated by oxidative stress [98].

## 6. Common Heavy Metal Pollutants

### 6.1. Lead

Lead is one of the most persistent environmental toxins implicated in cardiovascular toxicity today [120]. While public policy changes since the 1970s banning the use of lead in motor fuels, paints, and other common construction materials have meant an overall reduction in exposure to this harmful metal, the levels of lead in the environment still remain troublingly high [4]. Specifically, over 26 million people globally are still at risk of lead toxicity, leading to a loss of an estimated 9 million combined years [1]. In high-income countries like the US, lead exposure primarily occurs via sources such as aging lead piping, unreplaced lead paint in older houses, and leaded gasoline. For low-income countries, the exposure mainly comes through occupational hazards in industries within these countries, such as battery manufacturing, mining, and recycling plants. Beyond occupational exposure, lead is also heavily found in the soil and water supplies of these countries, adding risk to citizens [1,32]. Specifically, in terms of the methods of action, lead is absorbed via the respiratory and gastrointestinal tracts as well as occasionally through the skin. Once in the blood, almost 99% of the lead is rapidly bound by erythrocytes, after which 95% is moved into the bones [32]. This increases the stability of the lead significantly and serves as a consistent reservoir of toxicity even decades after exposure.

In terms of its specific cardiovascular effects, increased lead levels have been implicated in a variety of physiological changes, such as the impaired regulation of cardiac excitability and contractility, damaged endothelial and vascular function, dyslipidemia, impaired mitochondrial function, and increased inflammation [8]. These mechanistic changes can then significantly increase the risk of clinical pathologies such as hypertension, ischemic heart disease, stroke, congenital heart disease, and peripheral vascular disease [121,122,123,124,125]. A recent study used approximately 40,000 participants’ data from the United States National Health and Nutrition Examination Survey from 1999 to 2021 as representative of the US population. Tsoi et al. uncovered that doubling blood lead levels, even within the range considered acceptable, was associated with higher blood pressure when controlling for factors such as age, gender, ethnicity, waist circumference, poverty to income ratio, education, ever cigarette smoking, diabetes and stage 3–5 chronic kidney diseases [126]. This is especially concerning since just an increase of 2 mmHg in systolic blood pressure is associated with an increased risk of stroke [127]. Several other studies have corroborated this association, suggesting that high blood lead levels are associated with an increased risk of hypertension [128,129,130,131,132,133,134].

While the exact mechanism by which increased lead exposure causes hypertension is not well understood, researchers have suggested several physiological pathways may be involved. Chief amongst these is lead’s role in producing ROS through various mechanisms, including ETC complexes, NOX, and XO [30,31]. Lead also reduces SOD, CAT, and glutathione peroxidase (GPx) activities. Furthermore, lead elevates cytosolic Ca^2+^, activating protein kinase C (PKC) and phospholipases that amplify ROS generation [30].

**Figure 5:**
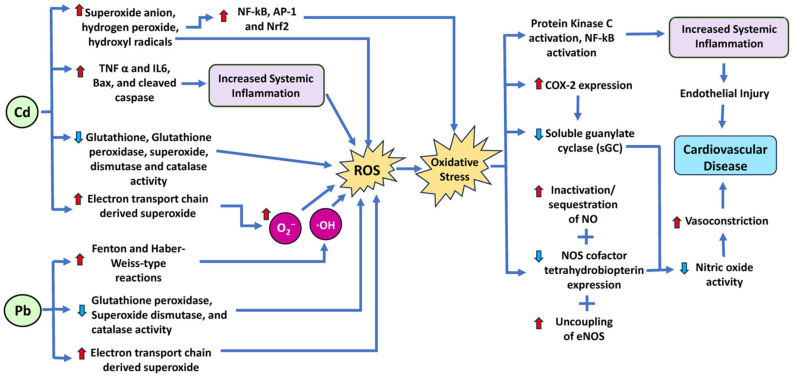
Schematic diagram of the mechanism and signaling pathways by which the heavy metals lead (Pb) and cadmium (Cd) increase cardiovascular disease risk. Both lead and cadmium induce ROS by various mechanisms, resulting in oxidative stress. IL, interleukin; TNF-α, tumor necrosis factor-α; Nrf2 nuclear factor erythroid 2-related factor 2; AP1, activator protein-1; NF-kβ, nuclear factor-kappa B. Red arrows indicate increased levels of that component, while light blue arrows show decreased levels of that component. The dark blue arrows indicate the direction of signaling.

Specifically, ROS, like superoxide (O_2_^−^) and hydrogen peroxide (H_2_O_2_), are unstable, highly reactive molecules usually created during the process of oxygen metabolism. When excessive generation of ROS happens, significant physiological tissue damage, such as tissue breakdown and activation of redox-sensitive transcription factors, occurs. Several prior studies have also implicated ROS in the pathogenesis of hypertension and other CVDs [135,136,137,138,139,140,141,142]. Research has demonstrated that lead participates in Fenton- and Haber-Weiss-type reactions, which promote the generation of ROS (Figure 5). Specifically, lead upregulates the Fenton and Haber–Weiss reactions, in which H_2_O_2_ is the substrate. During the reaction, H_2_O_2_ is converted to ·OH, which is highly toxic. Overproduction of ROS can then lead to significant oxidative stress within the cell [57,143]. Increased oxidative stress acts by limiting the bioavailability of active NO (a known vasodilator) via the inactivation/sequestration of NO by ROS, depletion of the NO synthase (NOS) cofactor tetrahydrobiopterin, and uncoupling of endothelial NOS (eNOS) [144]. Diminished NO in the heart results in the development of hypertension and CVDs. Furthermore, most actions of NO require the activation of soluble guanylate cyclase (sGC). Courtois et al. demonstrated that incubating normal rat aorta in a lead-containing medium for 24 h leads to a concentration-dependent downregulation of sGC expression, elevation of O_2_^−^ production, and upregulation of cyclooxygenase-2 (COX-2) expression [32,145]. Additionally, oxidative stress also leads to the generation of peroxynitrite (ONOO^−^), which is a highly toxic reactive nitrogen species and can contribute to cardiovascular, renal, and neurological damage [32]. Beyond direct oxidative stress, previous studies have also demonstrated that lead exposure caused protein kinase C activation, NF-κB activation, and angiotensin-converting enzyme (ACE) activity as a possible means of increasing the risk for hypertension [32]. Figure 5 shows the molecular mechanisms and signaling pathways by which lead and cadmium increase cardiovascular disease risk.

### 6.2. Cadmium

Another key environmental determinant of cardiovascular risk is cadmium (Cd). Cadmium is a toxic heavy metal that has been linked with a variety of pathologies, including cancer, reproductive damage, and renal damage. Exposure to this toxin can occur through a variety of sources, including contaminated air and water, as well as the byproducts of fossil fuels. Leaking sewage waste containing cadmium compounds is absorbed by plants, introducing them into the food chain, where they proceed to accumulate in various organs [146]. Presently, tobacco smoke is seen as the primary source of cadmium exposure, with research demonstrating that smokers have 4–5 times higher Cd levels in their blood compared with non-smokers [146]. Further industrial sources of cadmium include nickel–cadmium batteries, pigments in paint production, electroplating, and polyvinyl chloride plastic [146]. A central reason for concern with cadmium is its long retention times in human systems. Specifically, cadmium demonstrates a clearance half-life of 25 years and can be retained in organs such as the kidneys [147], bone, liver, lung, central nervous system, and cardiovascular system [148]. The primary means of entry for cadmium include inhalation and ingestion. Approximately 10–50% of inhaled Cd dust is absorbed, and 5–10% of ingested Cd is absorbed into the body [148].

In terms of CVD, Cd has been implicated in several pathologies, including hypertension, promotion of atherosclerosis, and impaired cardiac function [51]. While causal links have not been established between Cd exposure and certain pathologies, epidemiological evidence has shown that people living in areas of greater Cd exposure have a higher risk of cardiovascular mortality [149,150]. Furthermore, amongst the various CVDs in which Cd is implicated, the most common are cardiomyopathy and dilated cardiomyopathy. A cohort study of 3348 American Indian adults aged 45–74 years in the US measured urine Cd levels using inductively coupled plasma mass spectrometry to determine the association of urine Cd levels with CVD occurrence and mortality. The authors identified 1084 cardiovascular events, including 400 deaths, in their cohort study and demonstrated a significant association between urinary Cd and increased incidence of heart failure [151,152]. The exact pathway of Cd-induced cardiotoxicity remains unclear. However, ROS is a key mediator of cardiotoxicity [153,154]. Previous reports highlighted that Cd exposure induces oxidative stress in cells due to the accumulation of ROS and increases in lipid peroxidation and malondialdehyde (MDA), causing oxidative damage [155,156].

It is known that Cd leads to the uncoupling of oxidative phosphorylation, possibly by interacting with the Q-site of Complex I or other NADH-dependent enzymes, lowering their activity [157]. Prior work by Hirst has also demonstrated that inhibition of the Q-site significantly increases ROS production by Complex I [158]. This Complex I activity also leads to electrons accumulating in the Q-site, which can then be transferred to molecular oxygen, resulting in O_2_^•−^ generation [159]. In addition to ROS production via ETC complexes, Cd also induces ROS through NOX and increases ROS levels via the suppression of SOD, CAT, glutathione peroxidase (GPx), and GST activities (Figure 5) [47,48,49,160]. Cd also increases cytosolic Ca^2+^, activating phospholipases and ROS overproduction [50].

Furthermore, Cd causes changes in mitochondrial membrane permeability, releases cytochrome C, and induces mitochondrial caspase-dependent apoptotic protein expression through a cascade reaction, which can lead to apoptosis [161]. A study conducted in rat hearts showed that Cd treatment upregulated the expression of proinflammatory cytokines (TNF-α (tumor necrosis factor-alpha) and IL-6 (interleukin 6)), and apoptotic stimulants (Bax and cleaved caspase-3) in cardiac tissue [160]. Studies have also shown evidence of Cd-generated superoxide anion, H_2_O_2_, and hydroxyl radicals, which also activate sensitive transcription factors like NF-κB (nuclear factor kappa-light-chain-enhancer of activated B cells), AP-1 (activator protein-1), and Nrf2 (nuclear factor erythroid 2-related factor 2), thereby altering ROS-related gene expression [31]. Nrf2 is a transcription factor that regulates the expression of many antioxidant and detoxifying enzymes, such as glutathione peroxidase, catalase, SOD, heme oxygenase-1, and NAD(P)H/quinone oxidoreductase 1 [162]. Nrf2 can contribute to cardiovascular disease by inducing undesirable effects on vascular function and remodeling, such as by increasing the expression of xanthine dehydrogenase (XDH), which converts hypoxanthine and xanthine into uric acid and superoxide anion. Uric acid can induce vasoconstriction and inflammation, while superoxide anion can react with NO and impair its vasodilatory and antiplatelet effects [163].

Both lead and cadmium can bind to the heme group of eNOS, inhibiting it and decreasing NO production, resulting in impaired vasodilation and increased blood pressure. ROS can also deplete the cofactor tetrahydrobiopterin (BH4), which is essential for eNOS function and stability [164]. When BH4 levels are low, eNOS becomes uncoupled and produces more O_2_^−^ instead of NO, further exacerbating oxidative stress and endothelial dysfunction. Therefore, lead and cadmium can impair the NO pathway and cause oxidative stress by both direct and indirect mechanisms.

## 7. Household Toxins

Parabens are preservatives that are rampant in households and found in haircare products, cosmetics, foods, and even drinking water due to their ubiquity in products [165]. There is ample evidence that parabens are endocrine-disrupting chemicals (EDCs), demonstrated by parabens’ alteration of estrogen receptors, thereby modifying estrogens’ actions. Estrogen receptors regulate cardiac gene expression. Therefore, efforts were previously performed to explore the relationship between CVD and paraben exposure. Zhou et al. found that single-nucleotide polymorphisms (SNPs) in estrogen receptor genes 1 and 2 interacted with paraben to increase hypertension risk. Men were more susceptible to the hypertensive effect of paraben exposure than women, even though women had higher urinary concentrations of parabens from their hospital-based case–control study involving 396 hypertension cases and 396 controls in Wuhan, China [165]. The authors found a positive association between parabens and hypertension, with an additive and interactive effect contributed by SNPs on estrogen receptor genes [165].

Fan et al. found that ethyl paraben exposure caused abnormal cardiac function and heart morphology, mainly manifested as pericardial effusion and abnormal heart rate in the early-stage development of zebrafish embryos. Another interesting finding was that zebrafish heart rates showed a compensatory change in response to ethyl paraben in the early stage of cardiac function decline, where it first increased, then decreased, or stopped. Surprisingly, RNA-seq analysis performed to explore the molecular mechanism underlying the toxic effects of ethyl paraben in zebrafish uncovered that retinoic acid signaling, cardiac muscle contraction, and cell apoptosis pathways in ethyl paraben-exposed larvae were significantly influenced [98].

Parabens appear to contribute to the development of oxidative stress in plasma and erythrocytes [166]. Pollack et al. found that several parabens were associated with modestly reduced levels of erythrocyte GPx, while other parabens were associated with a decrease in SOD. Interestingly, they found that three parabens were related to increases in antioxidant enzymes. A review article of 27 studies from 2008 to 2019 examining the association between bisphenol-A (BPA) exposure and oxidative stress biomarkers found a positive association with the biomarker 8-Oxo-7,8-dihydro-2′-deoxyguanosine (8-OHdG), an indicator of DNA/RNA damage [167]. Another common household toxin is BPA, found in epoxy resins that coat water supply pipes and metal food cans, and polycarbonate plastics used to make water bottles or plastic containers. Moon et al. conducted a population-based analysis to find an association between BPA and CVD. When accounting for differences in urine dilution between 9265 participants using the natural log ratio of BPA to creatine (ln-BPA/Cr), higher levels of ln-BPA/Cr were associated with an increased risk of CVD, including ischemic heart disease and congestive heart failure [168]. Several in vivo studies in rats suggest that BPA-induced arrhythmia disrupts intracellular calcium signaling in cardiomyocytes and triggers atherosclerosis [63,64].

Oxidative stress has been suggested to be a contributing factor in paraben and bisphenol association with CVD. A case–control study of patients from Guangdong Provincial People’s Hospital in Guangzhou, China, found that butylparaben, BPA, and BPF significantly increased the risk of coronary heart disease [169]. Moreover, reduced high-density lipoprotein (HDL) and oxidative DNA damage mediated the increased CHD risk when exposed to bisphenols. Though HDL was a more significant mediator than oxidative stress, HDL is a scavenger of ROS, hinting at another relationship between oxidative stress and CHD. To draw any robust conclusions, further research is needed.

Another ubiquitous group of phenols is chlorophenols, which are used in industrial products and are formed as degradation byproducts from sewage and drinking water chlorination [170]. 2,5-dichlorophenol (DCP) is often used in room and toilet deodorizers and for the synthesis of resins and dyes. Using data from a nationally representative survey, the National Health and Nutrition Examination Survey, Rooney et al. found that higher urinary concentrations of 2,5-DCP were associated with higher rates of CVD and all forms of cancer in a sample of 3617 adult participants [171]. Mechanistically, Li et al. found that 2,4-DCP decreased ATP production and mitochondrial respiration in fathead minnow embryos, causing developmental toxicity, while heart rate was significantly decreased [172].

Phthalates are also a common group of domestic toxins. According to the FDA’s 2010 Survey of Cosmetics for Phthalate Content, phthalates were found in some nail polishes, body lotions, fragrances, deodorants, hair care products, body washes, and more (U.S Food and Drug Administration, 2022 [173]). Also of note is that the FDA does not require cosmetic products and ingredients to be approved and does not currently suggest that phthalates in such products pose a health risk. A common phthalate, Di(2-ethylhexyl) phthalate (DEHP), is found in plastic products such as tablecloths, shower curtains, garden hoses, toys, and swimming pool liners.

When mice were treated with DEHP daily for 28 days, DEHP induced cardiac mitochondrial damage [174]. In another study, mice were injected via an intragastric route with DEHP daily for 28 days, leading to cardiomyocyte mitochondrial damage and thus oxidative stress [175]. A review of studies on the relationship between DEHP and CVD found that DEHP increases the development and severity of CVD due to oxidative stress and inflammation [176]. Alongside BPA and phthalates, perfluorinated compounds (PFCs) are added to plastics during processing and make their appearance in the food chain, namely, via plastic degradation [177]. The perfluorinated compound perfluorooctane sulfonate (PFOS) has been detected in drinking water, soil, air, dust, and sediment, and due to its high chemical stability, humans ingest it in food and water often enough to result in measurable blood serum levels [178]. It has been previously shown that PFOS might be linked to the development of CVD, particularly atherosclerosis, leading Wang et al. to treat mice with the dose-equivalent of the human tolerable daily intake of PFOS. They found that chronic PFOS exposure increases atherosclerosis progression by polarizing M1 macrophages, inducing proinflammatory cytokines, and contributing to the progression and instability of arterial plaques, making them more likely to rupture [178]. In line with these findings, Xu et al. found that rats that were injected intraperitoneally with PFOS suffered from significant myocardial injury, myocardial apoptosis, and cardiac inflammation [179]. Cardiovascular damage from PFOS extends beyond the immune system, additionally impairing the structure and function of mitochondria in mouse embryonic stem-cell-derived cardiomyocytes [180]. PFOS disrupted calcium fluxes and the mitochondria-associated endoplasmic reticulum structure and decreased ATP production and cardiomyocyte differentiation. Furthermore, the Rictor/mTORC2 signaling pathway was activated by PFOS and found to be responsible for the mitochondria-associated endoplasmic reticulum membrane (MAM) structure impairment, altered fatty acid metabolism and glycolysis, and disrupted mitochondrial physiology [180]. In zebrafish larvae, the PFC perfluorotetradecanoic acid (PFTeDA) induced the upregulation of several antioxidant enzymes and genes associated with Complex I and IV of the electron transport chain to combat ROS production and maintain oxidative respiration [181]. In contrast, when Liu et al. treated zebrafish with significantly higher concentrations of perfluorononanoic acid, ROS generation increased, and mitochondrial- and oxidative stress-related genes were downregulated [182]. This indicates a dose-dependent relationship as well as a significance in the minor chemical structure differences between PFCs, where, for instance, mitochondrial membrane permeability and proton leak were observed with perfluorooctanoic acid (PFOA) but not with PFTeDA [183]. Similar findings of ROS generation in response to exposure to PFCs have been demonstrated in human cells. When immortalized primary human microvascular endothelial cells were treated with both high and low concentrations of PFOS, ROS production increased [184]. The low concentrations of PFOS used were both occupationally and environmentally relevant, indicating that ROS generation may occur at typical human exposure ranges.

## 8. Mycotoxins

**Figure 6:**
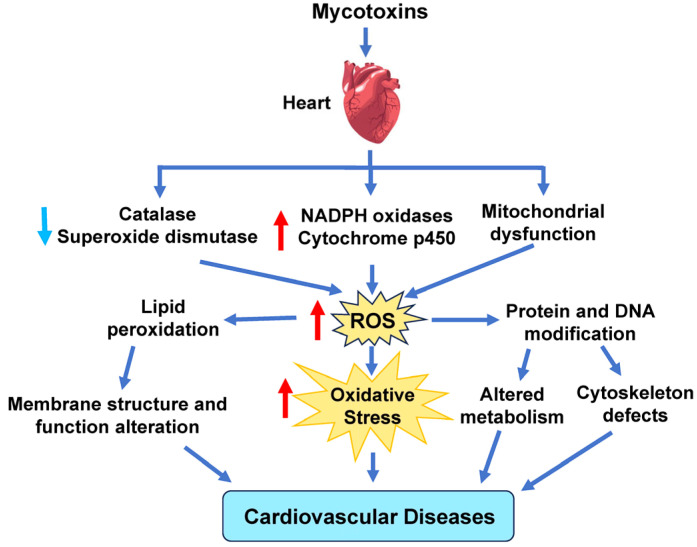
Effect of mycotoxins on cardiovascular disease. Mycotoxins can cause elevated ROS production by decreasing catalase and superoxide dismutase activity and increasing ROS derived from NADPH oxidases, cytochrome P450, and the mitochondrial electron transport chain. Red arrows indicate increased levels of that component, while light blue arrows show decreased levels of that component. The dark blue arrows indicate the direction of signaling.

Mycotoxins are toxic secondary metabolites produced by mold and fungi that can contaminate many kinds of food and sprout in buildings damaged by water [185,186]. Even animal byproducts, such as milk and eggs, can also be contaminated with mycotoxins through animals ingesting feed contaminated with mycotoxins [187]. Exposure to mycotoxins can lead to increased amounts of ROS in the body, leading to oxidative stress and cellular damage (Figure 6). This increase in ROS levels is due to enhanced ROS production by NOX, CYP, and ETC complexes. Reduced GSH levels and SOD and CAT activity further raise ROS levels [93,94,95].

Even low levels of mycotoxins can result in inflammation of the vasculature, disruption of lipid metabolism, breakdown of osteocytes for calcium, and obstruction of the arteries. Prolonged inflammation can lead to more adverse effects, such as endothelial dysfunction, vascular oxidative stress, lipid peroxidation injury, and vascular lesions [185].

Aflatoxin is a well-recognized type of mycotoxin known for being the most toxic and abundant carcinogen [186]. The presence of aflatoxin in the body causes activation of cytochrome P450, leading to increased ROS production and Toll-like receptor 4 (TLR-4) regulation. The increased upregulation of TLR-4, an inflammatory mediator, results in an intensified immune response. Aflatoxin is also capable of disrupting metabolism, including the dysfunction of the Krebs cycle, glycolysis, and mitochondria [185]. This effect, in combination with the high NO concentration from the endothelial dysfunction and the increased inflammatory response, can result in apoptosis of cardiomyocytes. This effect was observed during an experiment that involved exposing rats to low concentrations of aflatoxin. The rats’ myocardium exhibited areas of necrosis, inflammation, and a decrease in cardiomyocytes, resulting in cardiac damage [185,188].

## 9. Pharmaceutical Products

Pharmaceuticals are generally used for their therapeutic, preventive, and diagnostic properties since they play a vital role in human health outcomes. There has been a continuous increase in the global use of pharmaceuticals and personal care products (PPCPs) in the last decade due to advances in research and development, the growing world population, and increased accessibility to healthcare and pharmaceuticals [189]. Approximately 100,000 over-the-counter (OTC) medicines and personal care products are sold in pharmacies and convenience stores in the US alone [189]. Inevitably, PPCPs are released into the environment at different phases of their lifecycle, like many other man-made chemicals. In recent years, PPCPs have been of great concern due to their detection, even at low concentrations (picograms per liter, pg/L), in sewage, surface waters, groundwater, drinking water, soil, and aquatic organisms [189]. The toxicity and concentration of PPCPs in waste treatment plants (WTPs) and water bodies are increasing day by day due to the increased rate of indiscriminate and mass consumption of these organic compounds [190]. Surprisingly, one of the most used pharmaceuticals that few think about with respect to causing cardiotoxicity is non-steroidal anti-inflammatory drugs (NSAIDs), such as ibuprofen [97,191]. Therefore, this section explores the potential health implications of NSAIDs, such as ibuprofen exposure, to the cardiovascular system.

### 9.1. Ibuprofen

NSAIDs (notably ibuprofen) are among the top 10 persistent pollutants, accounting for more than 15% of all pharmaceuticals detected in aquatic environments [192]. Ibuprofen metabolites (carboxy-ibuprofen, hydroxy-ibuprofen, and ibuprofen–acyl-glucuronide conjugates) are more toxic and may potentially pose a greater risk than its parent molecule [190,193]. Ibuprofen concentrations in influent wastewater have been recorded to reach between 5.78 and 1673 mg/L [194]. Due to the presence of ibuprofen or its metabolites in the environment, they are consumed by plants and aquatic organisms, allowing ibuprofen and its metabolites to enter the food chain [190]. The bioaccumulation of ibuprofen and other NSAIDs in aquatic organisms, like fish, mollusks, and crustaceans, can also lead to their transfer and concentration further up the trophic chain [190,195]. The concentrations of ibuprofen and its metabolites in wastewater signify an ecological hazard and can act as a source of adverse environmental effects because ibuprofen’s stable chemical structure and biological activity make it highly resistant to biodegradation, thereby resulting in its toxic and persistent presence in human and animal food chains [194,196].

Even though the direct effects of these pharmaceutical pollutants on aquatic organisms are certain, their resulting impact on human health and well-being is challenging to measure accurately. Nevertheless, orally and topically administered NSAIDs have the potential to cause a wide range of direct negative side effects on human health, including increasing the risk of adverse events in the cardiovascular system (MI, heart failure, and hypertension) [97]. An ecological risk assessment study conducted in a French national survey found that the estimated real risk ratio of ibuprofen was ≤1, suggesting that it was an environmentally risky substance [197]. Moreover, the effects of ibuprofen are most likely dose-dependent and rely on the degree of exposure—acute or chronic. Female B6C3F1 mice treated with 100, 200, and 400 mg/kg of ibuprofen for 2 weeks had significantly increased liver weights. Tiwari et al. found that ibuprofen resulted in sex-specific changes in energy metabolism and protein degradation pathways in livers of mice treated with a moderate daily dose of ibuprofen for 7 days [198]. This implies that ibuprofen has the potential to affect males and females differently.

### 9.2. Mechanism and Signaling Pathways by Which NSAIDs Increase Cardiovascular Disease Risk

Several clinical studies have been conducted in the past few years to ascertain the safety and effectiveness of NSAIDs in CVD. It was found that the risk of atrial fibrillation, heart failure, MI, and other cardiovascular conditions increased in patients with a history of these pathological conditions [97,199]. Both nonselective NSAIDs (e.g., ibuprofen) and selective NSAIDs result in the development of hypertension in both normotensive and hypertensive individuals, and NSAIDs use interferes with antihypertensive medications except for calcium channel blockers [97]. Additionally, the authors reported that the use of NSAIDs with other antiplatelet drugs (except aspirin) increased the rate of cardiovascular events like cardiovascular death, MI, and stroke [97,200].

The biological mechanism for the NSAID-associated risk of MI has primarily emphasized pro-thromboembolic effects, but NSAIDs have also been found to influence renal function and the regulation of fluid balance, causing fluid retention and worsening of heart failure, all of which contribute to the risk of MI [201]. Furthermore, NSAIDs have been found to interact with antihypertensive drugs, such as angiotensin-converting enzyme inhibitors, through mechanisms related to the inhibition of prostaglandin synthesis, which interferes with the renal vasculature and the regulation of blood pressure [202]. Moreover, NSAIDs can increase serum aldosterone levels, leading to sodium retention and hypertension [191,203]. NSAIDs have also been associated with an increased risk of atrial fibrillation [191]. The biological mechanism involved is not well understood, but it is suggested to involve adverse effects on fluid retention, serum electrolytes, and blood pressure [201,204,205].

**Figure 7:**
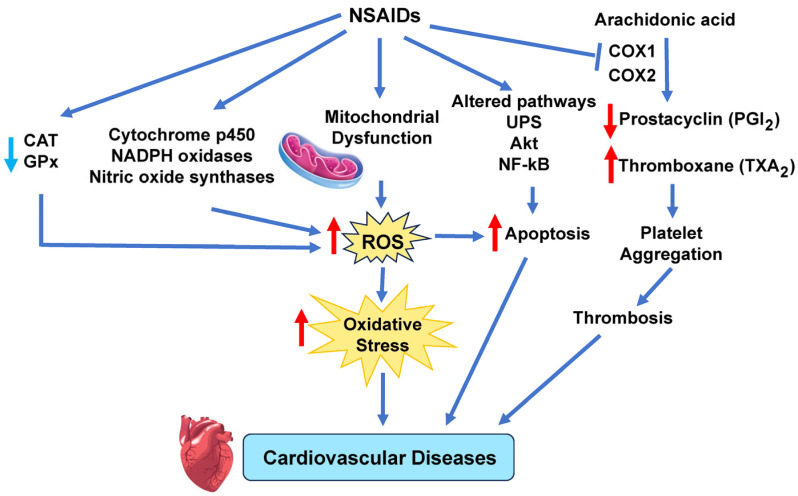
NSAID induce cardiovascular disease. NSAIDs induce mitochondrial dysfunction and alterations in several enzymes that increase ROS. Elevated ROS levels disrupt cellular redox balance, overwhelming antioxidant systems, and creating oxidative stress. Prolonged oxidative stress damages blood vessels and heart tissue, leading to different cardiovascular diseases. Akt, protein kinase B; CAT, catalase; COX, cyclooxygenase; GPx, glutathione peroxidase; NF-kB, nuclear factor kappa-light-chain-enhancer of activated B cells; UPS, ubiquitin–proteasome system. Red arrows indicate increased levels of that component, while light blue arrows show decreased levels of that component. The dark blue arrows indicate the direction of signaling.

The main players implicated by several studies in NSAID-induced cardiotoxicity are the generation of ROS in the mitochondrial ETC, NOX, XO, and lipoxygenase, cyclooxygenases, NOS, and cytochrome P450-based enzymes [97]. ROS levels are further elevated due to decreased CAT and GPx activities [97]. As a result, several other cell signaling pathways (p53, Vax, Bcl-2, mitochondrial Cyt C, NF-KB, apoptosis-inducing factor, Akt, and ubiquitin–proteasome system) that can increase the production of ROS occur, leading to platelet aggregation and thrombosis, acute MI, apoptosis, reperfusion injury, and heart failure due to prolonged oxidative stress in the heart (Figure 7) [97]. Specifically, Li et al. found that four different types of NSAIDs, diclofenac, naproxen, rofecoxib, and celecoxib increased NADPH oxidase expression, eNOS expression, and nitrite levels in the aorta and heart of spontaneously hypertensive rats model, concluding that the aorta and heart of the NSAID-treated rats showed increased O_2_^•−^ production and oxidative stress [206]. In vitro studies by Ghosh et al. showed that at pharmacological levels, the NSAID meclofenamate sodium induced ROS and inhibited mitochondrial respiratory complexes [207]. Several studies have also shown that ibuprofen increased ROS levels which induce lipid peroxidation and DNA damage and increased expression of endogenous antioxidant systems such as SOD, catalase (CAT), and glutathione peroxidase (GPx) [208,209].

One potential way that NSAIDs may be causing cardiovascular disease is via decreased proteasomal activity in cardiac cells. Our laboratory has found that NSAIDs and pesticides decrease proteasome activity [57,207,210]. Proteasome dysfunction has been implicated in the pathogenesis of various cardiovascular diseases, such as atherosclerosis, myocardial ischemia–reperfusion injury, hypertrophy, heart failure, and arrhythmias [211]. The decreased proteasome activity observed will likely result in an accumulation of damaged or misfolded proteins in cardiac cells, which can trigger cellular stress responses, inflammation, apoptosis, and fibrosis. However, since the proteasome is so fundamental to survival, many signaling pathways are likely to be involved (such as the NF-κB pathway) that need to be investigated.

Endothelial cells play a critical role in maintaining vascular homeostasis, which is essential for the control of many cardiovascular diseases, including atherosclerosis and thrombosis [97,212]. Interestingly, Liou et al. [213] found that 160 μM sulindac (an NSAID) induced endothelial apoptosis in human umbilical vein endothelial cells (HUVECs), as evident by an increase in annexin V-positive cells. Both sulindac and indomethacin significantly increased cleaved poly(ADP-ribose) polymerase (PARP) levels, as well as increased the level of the apoptotic activating factor caspase-3 [213]. The apoptosis in HUVECs induced by the NSAIDs was associated with reduced PPARδ and 14-3-3-ε expression. Under normal conditions, 14-3-3-ε binds phosphorylated Bad, inhibiting the translocation of Bad to the mitochondria and preventing apoptosis through the mitochondrial pathway. However, sulindac, through the suppression of 14-3-3-ε expression, increased Bad translocation to mitochondria, thereby inducing apoptosis [97,213].

## 10. Microplastics

Plastic is a globally produced material used in many common industries, such as the automobile and home goods industries. The high amounts of plastics present throughout the world lead to a higher accumulation of plastic in the environment, which then degrades into microplastics and nanoplastics. Recent studies show that higher exposure to microplastics and nanoplastics leads to harmful effects in human and animal cell models, which include the cardiovascular system phenotype [214]. Several CVD risk factors are associated with higher microplastic and nanoplastic exposure, mainly through the skin, mouth, and lungs. In a review by Zhu et al., the associated risk factors found were myocardial fibrosis, altered heart rate, abnormal blood velocity, decreased cardiac output, endothelial dysfunction, and cardiometabolic disease phenotype [214]. The degraded microplastics and nanoplastics can result in particles small enough to pass through the intestinal epithelium blood vessels, the pulmonary blood–air barrier, and macrophage cells. It was found that microplastics and nanoplastics can cause multiple adverse cardiovascular effects through various means, including oxidative stress, inflammation, apoptosis, pyroptosis, and cellular interaction, leading to the risk of CVD. Several studies found that, depending on the size of the plastic, nanoplastics can accumulate in the blood, and microplastics can accumulate in various organs in rodent models [214].

Studies also found that offspring in animal models could be exposed to microplastics and nanoplastics through the placenta and the mother’s milk [214,215]. There were also studies that analyzed the effect of microplastics and nanoplastics on other environmental hazards. One study found that a combination of micro- and nanoplastic exposure with chloroauric acid hydrate resulted in an increase in the rate of mortality, underdevelopment of the heart, and development of yolk edema in a zebrafish model [214,216]. Another study using zebrafish found that the combined effects of 2,2′,4,4′-tetrabromodiphenyl ether (a flame retardant) and microplastics and nanoplastics caused a decreased heart rate and pericardial edema. However, micro- and nanoplastics also resulted in protective effects against some environmental toxins. Zebrafish larvae that were exposed to polycyclic aromatic hydrocarbons (PAHs), a type of carcinogenic pollutant, and micro- and nanoplastics displayed a protective effect from the plastic against the toxic cardiovascular effects of the PAHs. Micro- and nanoplastics exhibited a protective response when zebrafish larvae were subjected to PAHs, mitigating the detrimental cardiovascular effects of the PAHs. The main contributor to these protective effects from micro- and nanoplastics seems to be that the plastics and the other environmental toxins compete against each other, reducing the amount of toxins able to enter and accumulate in the embryos [214]. It should be noted that many microplastic studies test doses of micro- and nanoplastics that were much higher than the concentrations found in the environment, making it difficult to determine whether the effects noted are realistic for actual situations [214,217].

**Figure 8:**
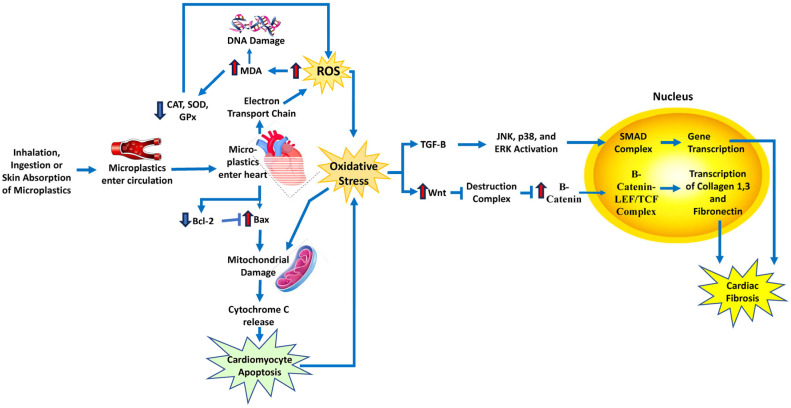
Schematic diagram of the mechanism and signaling pathways by which microplastics increase cardiovascular disease risk. Microplastics induce ROS in heart tissue, leading to oxidative stress and activation of pathways involved in cardiac fibrosis. Bax, BCL2-associated X, apoptosis regulator; Bcl2, B-cell lymphoma 2; LEF/TCF, lymphoid enhancer-binding factor/T-cell factor; JNK, c-Jun N-terminal kinase; MDA, malondialdehyde; SMADs, sma- and MAD-related proteins; TGF-β, transforming growth factor-β. Red arrows indicate increased levels of that component. The dark blue arrows indicate the direction of signaling.

A study by Li et al. exposed rats to various concentrations of polystyrene microplastics in their drinking water for ninety days [112]. Researchers found that when the microplastics are small enough, they can pass through the epithelial cells in the stomach and enter the circulation, where the microplastics can then enter other organs in the body. Two essential markers of myocardial damage were found to be significantly elevated in rat hearts: creatine kinase-MB and troponin I. The lipid peroxidation marker, malondialdehyde (MDA), was significantly elevated in the rat hearts. This is likely due to the microplastics-induced elevated ROS production by mitochondrial ETC complexes [110,111]. Additionally, several antioxidants, CAT, GPx, and SOD, were also significantly decreased, demonstrating that oxidative stress occurred in the hearts of the rats that ingested microplastics (Figure 8). In these treated mice, Bcl-2, a protein that inhibits apoptosis, was downregulated, and Bax, a protein that stimulates apoptosis, was upregulated, suggesting that cardiomyocyte apoptosis occurs in rat hearts due to oxidative stress and mitochondrial damage [112].

Oxidative stress can also trigger the Wnt pathway, which is responsible for the production of myofibroblasts, leading to the development of cardiac fibrosis. In the rat hearts, there was an increase in Wnt, the first molecule in the pathway that activates signals in the cell, and an increase in β-catenin, which forms a complex once in the nucleus of the cell to activate gene transcription. This resulted in the increased production of collagen 1, collagen 3, and fibronectin, which leads to fibrosis (Figure 8). The myofibroblast marker α-SMA and the cardiac fibrosis marker TGF-β were also significantly increased in the rat hearts, showing that oxidative stress, which was caused by the ingestion of polystyrene microplastics, resulted in the development of cardiac fibrosis in the rat hearts [112].

## 11. Clinical Implications of Oxidative Stress

Antioxidant therapy: The administration of exogenous antioxidants, such as vitamins C and E, beta-carotene, coenzyme Q10, resveratrol, or polyphenols, that can scavenge ROS and protect biomolecules from oxidative damage has been significantly investigated. However, the efficacy and safety of antioxidant therapy for cardiovascular diseases are controversial, as some studies have shown beneficial effects, while others have shown no effect or even adverse effects [218,219,220]. These controversial effects of antioxidant therapy are not exclusive to cardiovascular diseases [221]. Moreover, antioxidant therapy may interfere with the physiological roles of ROS, such as cell signaling and adaptation, and may reduce the endogenous antioxidant defense systems, resulting in a paradoxical increase in oxidative stress. An example of antioxidant therapy that had no effects is the HOPE and HOPE-TOO trials, which investigated the effects of vitamin E on the prevention of cardiovascular events and cancer in high-risk patients with vascular disease or diabetes [218]. The trial enrolled 9541 patients and randomly assigned them to receive either vitamin E (400 IU/day) or placebo for a mean duration of 7 years. The results showed that vitamin E had no significant effect on the primary endpoint of myocardial infarction, stroke, or cardiovascular death, nor the secondary endpoints of cancer, unstable angina, congestive heart failure, or revascularization. Moreover, vitamin E was associated with a higher risk of heart failure and hospitalization for heart failure. The authors concluded that vitamin E does not prevent cardiovascular events or cancer in high-risk patients and may be harmful.

Studies that show more beneficial effects include mitochondrial-targeted antioxidants and Nrf2 activators. Mitochondrial-targeted antioxidants have been shown to protect against atherosclerosis [222], ischemia/reperfusion (I/R) injury, heart failure, and diabetic cardiomyopathy in animal models and may offer a novel strategy for cardiovascular protection [223,224,225]. Nrf2 activators, such as sulforaphane, curcumin, or dimethyl fumarate, can enhance the cellular antioxidant capacity and protect against oxidative stress-induced cardiovascular injury [226]. Nrf2 activators and modulators have been shown to attenuate atherosclerosis, I/R injury, hypertension, and cardiac hypertrophy in animal models and may have therapeutic potential for human cardiovascular diseases [227,228].

**Figure 9:**
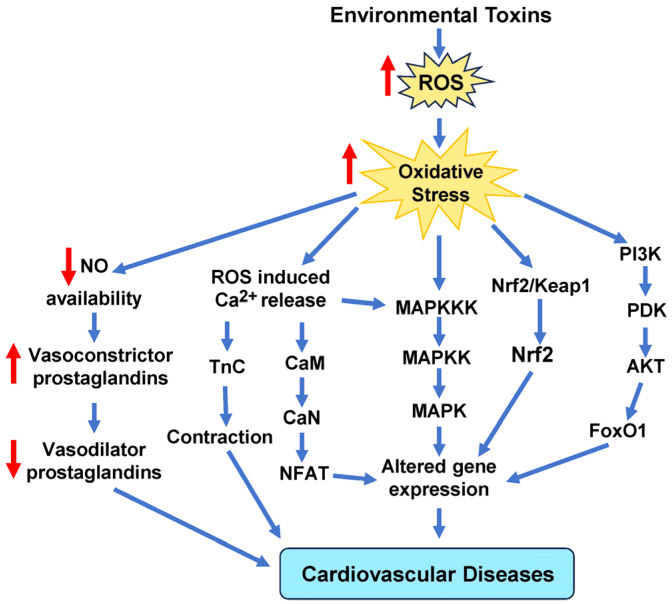
Schematic representation of the main signaling cascades in cells due to oxidative stress induced by environmental toxins. These signaling pathways include MAPKs, calcium, Nrf2, and NO signaling molecules that mediate signal transduction to alter gene expression that ultimately leads to cardiovascular disease. AKT, protein kinase B; CaM, calmodulin; CaN, calcineurin; FoxO1, forkhead box protein O1; MAPKKK, mitogen-activated protein (MAP) kinase kinase kinase; NFAT, nuclear factor of activated T cells; NO, nitric oxide; Nrf2, nuclear factor erythroid 2-related factor 2; PDK, pyruvate dehydrogenase kinase; PI3K, phosphoinositide 3-kinase; TnC, troponin c.

While some antioxidant therapies seem to have beneficial effects with respect to CVDs, some studies in mice models revealed that supplements with antioxidants, such as NAC, vitamin E, and the soluble form of vitamin E called Trolox, encouraged the growth and spread of tumors [229,230] (Le Gal et al., 2015, Zou et al., 2021). As more clinical trials are performed to understand the nuances associated with antioxidant therapy, individuals should be encouraged to eat more food rich in antioxidants. A study of the relationship between non-enzymatic antioxidant capacity (NEAC) from food and death from any cause or specific causes found that eating foods with higher NEACs was linked to a lower risk of death from any cause and death from CVD in Japanese adults [231]. None of the 42,520 men and 50,207 women who participated in this study had ever had cancer, stroke, ischemic heart disease, or chronic liver disease before. Figure 9 illustrates some of the key intracellular signaling pathways that are implicated in oxidative stress-induced CVD caused by environmental toxins.

## 12. Conclusions

The interplay between environmental toxins and oxidative stress is an expanding area of research that sheds light on previously underappreciated factors contributing to the burden of CVDs. While the mechanisms of action of different environmental toxins may partly depend on other factors, such as genetic susceptibility, nutritional status, or lifestyle habits, there is substantial evidence that oxidative stress is a common pathway through which many environmental toxins can cause cellular damage and dysfunction, leading to various diseases and disorders. A comprehensive understanding of CVD-related pathways altered by environmental toxins has the potential to inform public health policies, clinical interventions, and preventive measures aimed at reducing cardiovascular risks associated with environmental exposures.

## 13. Future Directions

Emerging research has unveiled a significant link between the gut microbiome and ROS production [232,233]. Certain bacterial species within the gut microbiome, particularly those involved in the fermentation of dietary components like fiber, produce metabolites that can modulate oxidative stress. Short-chain fatty acids, for instance, are byproducts of microbial fermentation and have been shown to possess antioxidant properties. They can help regulate ROS levels in the gut and influence systemic oxidative stress. When the gut microbiome experiences an imbalance in composition and function, it can lead to a proinflammatory state, which is linked to CVD. An imbalanced microbiota can promote the production of proinflammatory cytokines and other molecules that exacerbate oxidative stress in the vascular system. This chronic inflammation and oxidative stress are key contributors to atherosclerosis, the underlying cause of most CVDs. As such, understanding the intricate relationship between the microbiome, ROS, and CVD will likely offer new therapeutic avenues. In-depth research exploring the intricate interplay between an individual’s unique susceptibility and their genetic makeup is needed to unravel the nuanced mechanisms that underlie how environmental toxins exert their influence on oxidative stress and increase the risk of CVD. Such research will advance our understanding of the complex relationship between genetics, environmental factors, oxidative stress, and cardiovascular health.

## Figures and Tables

**Table 1:** Effect of environmental toxins on oxidative stress and cardiovascular disease.

Environmental Toxin	Enzymes and Mechanisms Involved in ROS Production	Effect on Cardiovascular Disease
**Air Pollutants**		
Particulate Matter (air pollutant)	Fine particulate matter in polluted air, such as Particulate Matter 2.5 (PM2.5) and PM10, can generate ROS in the respiratory tract when inhaled, by increased NADPH oxidases (NOX), increased leaking of electrons from the mitochondrial electron transport chains (ETCs), stimulation of immune cells that produce ROS, decrease of glutathione (GSH) levels, and decrease of catalase (CAT), glutathione S transferase (GST), and superoxide dismutase (SOD) activity [11,12,13]. Particulate matter can induce inflammation, which increases ROS production via cytokine signaling (e.g., tumor necrosis factor-alpha (TNF-α)) [14,15].	Induces cardiac dysfunction, increased cardiac injury, and acute myocardial infarction in Apolipoprotein E^−/−^ (ApoE^−/−^) mice [16].
		Increased blood expression, heart weight, cardiac expression of hypertrophic markers (ACTA1 (α-actin) and MYH7 (β-myosin heavy chain)), and decreased cardiac stroke volume and output in rats [17].
		Cardiac hypertrophy and fibrosis led to a decrease in cardiac systolic function in mice [18].
		Cardiac fibrosis in 4-week and 10-month-old mice. 10-month-old mice demonstrated cardiac diastolic dysfunction, elevated heart rate/blood pressure, and cardiac systolic dysfunction [19].
		Aggravates left ventricle failure-induced pulmonary hypertension in mice [20].
		Decreased cardiac growth, cardiac dysfunction, abnormal mitochondrial structure and function, and cardiac metabolic disorder [21].
		Neonatal and adult cardiac dysfunction [22].
		Oil Mist PM induces oxidative stress, inflammation, and mitochondrial dysfunction, inducing myocardial tissue injury [23].
		Polluted urban air aggravates myocardial infarction by enhancing mitochondrial H_2_O_2_ production and altering mitochondrial ultrastructure and function [24].
Ozone (O3) (air pollutant)	Ground-level ozone, a component of smog, can induce ROS in the heart by increasing the activity of NOX, increased leaking of electrons from the ETC and reducing SOD and CAT activity [25,26,27].	Platelet aggregation [28].
		Increased heart rate variability. Significant increase in the number of cardiac arrhythmias [29].
**Heavy Metals**		
Lead (heavy metal)	Lead stimulates ROS production through various mechanisms, including ETC complexes, NOX, and xanthine oxidase (XO) [30,31]. Lead reduces SOD, CAT, and glutathione peroxidase (GPx) activities. Lead elevates cytosolic Ca^2+^, activating protein kinase C (PKC) and phospholipases that amplify ROS generation [30].	Elevated arterial pressure [32].
		Increased systolic blood pressure, increased prevalence of left ventricular hypertrophy in humans [33].
		Left ventricular hypertrophy and alterations in cardiac rhythm [34,35].
		Hypertension—marked rise in systolic blood pressure [36].
Mercury (heavy metal)	Mercury exposure, especially in its methylmercury form found in contaminated fish, can lead to ROS production through various mechanisms, including ETC complexes and NOX. Mercury reduces GPx and thioredoxin reductase (TrxR) activity [37,38,39,40]. It stimulates Nitric oxide synthase (NOS) to produce nitric oxide (NO), which can form peroxynitrite, a potent oxidant [39,41].	Increased platelet aggregation and thrombosis [42].
		Increased systolic blood pressure [43].
		Increased arterial blood pressure [44].
		Hypertension, generalized atherosclerosis, coronary heart disease (CHD), myocardial infarction (MI), cardiac arrhythmias, heart rate variability, sudden death, cerebrovascular accidents (CVA), and carotid artery disease [45].
		Increased nonfatal ischemic heart disease (IHD), CVD mortality, and mortality due to other heart diseases [46].
Cadmium (heavy metal)	Cadmium (Cd) stimulates ROS production via ETC complexes and NOX. SOD, CAT, GPx, and GST activities are suppressed [47,48,49]. Cd increases cytosolic Ca^2+^, activating phospholipases and ROS overproduction [50].	Atherosclerosis and impaired cardiac function [51].
		Elevated blood pressure [52].
		Acute myocardial infarction and increased CHD mortality [53].
		Increased formation of atherosclerotic plaques [54].
**Pesticides**		
Organophosphates (pesticide)	Organophosphates can promote ROS production through various mechanisms, including ETC complexes, NOX, XO, cytochrome P450 (CYP)-induced ROS, and reduced SOD, GSH, CAT, and GPx activity [55,56,57].	High rates of developing arrhythmia, CAD, and congestive heart failure (CHF) [58].
**Endocrine-Disrupting Chemicals (EDCs)**		
Endocrine-Disrupting Chemicals	EDCs increase ROS by NOX and ETC complexes [59]. Changes in the unfolded protein response (UPR) due to endoplasmic reticulum (ER) stress increase ROS through Ca^2+^ release and disrupted protein folding. ROS levels are further elevated by lower GSH amounts and lower SOD, CAT, and GPx enzyme activities [60,61].	High-dose BPA exposure in fetal mouse heart development causes oxidative stress, mitochondrial dysfunction, and congenital heart defects [62].
		Bisphenol-A induced arrhythmia and triggered atherosclerosis in rats [63,64].
**Polychlorinated Biphenyls (PCBs)**		
Polychlorinated Biphenyls (PCBs)	PCBs induce ROS production by NOX, CYP, and ETC complexes [65]. Decreased SOD and GSH activities increase ROS levels [66].	Increased risk of hypertension [67].
		Elevated risk of hospitalization for CHD, acute myocardial infarction (AMI), and stroke [68].
		Increased heart failure risk [69].
		Increased rate of hypertension [70].
**Dioxins**		
Dioxin	Dioxins found in certain environmental pollutants, like the byproducts of waste incineration, can stimulate ROS production, leading to oxidative stress and various health issues. ROS levels are increased by CYP-derived ROS, decreased CAT and SOD activities, and lower GSH levels [71,72,73].	Increased incidence of degenerative cardiovascular lesions, including cardiomyopathy and chronic active arteritis in rats [74].
		Increased blood pressure and heart weight in mice [75].
		Increased blood pressure and development of severe atherosclerotic lesions in ApoE^−/−^ mice [76].
		Associated with mortality from ischemic heart disease (IHD); reduced blood supply to the heart [77].
		Induced dilated cardiomyopathy and myocardial hypoxia in avian embryos [78].
		Several forms of congenital cardiovascular malformations in humans [79].
		Cardiac hypertrophy and bradycardia in mice [80,81].
**Asbestos**		
Asbestos	Asbestos fibers can induce ROS production when inhaled, contributing to oxidative stress in lung tissues. ROS levels are increased by ETC complexes and decreased GSH levels [82,83].	Increased risk of cardiovascular-related diseases in exposed workers [84].
**Radon**		
Radon	Inhalation of radon causes increased ROS generation via radiation-induced ionization [85]. Radon disrupts ETC complexes and depletes SOD, resulting in increased ROS levels [86].	Radon accelerates low-density lipoprotein (LDL) accumulation, foam cell formation, and arterial thickening or fibrosis [85].
**Tobacco Smoke**		
Cigarette Smoke	Tobacco smoke contains a mixture of toxic chemicals, including ROS-generating compounds like free radicals and reactive carbonyls, which contribute to oxidative stress and are linked to smoking-related diseases. Increased ROS production comes from NOX, CYP, NOS, and ETC complexes. Lower levels of GSH, SOD, and CAT weaken antioxidant defenses, leading to increased ROS accumulation [87,88,89,90].	Atherosclerotic plaques in the carotid artery [91].
		Increases the risk of ischemic stroke [92].
**Mold and Indoor Air Contaminants**		
Mold Byproduct	T-2 mycotoxin increases higher ROS levels via NOX, CYP, and ETC complexes. Reduced GSH levels and SOD and CAT activity further raise ROS levels [93,94,95].	T-2 mycotoxin induces interstitial hemorrhage, capillary dilation, mitochondrial impairment, and fibrotic damage [96].
**Pharmaceuticals and Personal Care Products**		
Pharmaceuticals and Personal Care Products	NSAIDs such as naproxen and ibuprofen are associated with increased ROS production. This occurs by NOX, CYP, ETC complexes, and NOS, which contribute to peroxynitrite. ROS levels are further elevated due to decreased CAT and GPx activities [97].	Increased risk of stroke, hypertension, and coronary artery disease [97].
		Induced abnormal cardiac function and morphology (pericardial effusion) and abnormal heart rate in zebrafish embryos [98].
**Radiation**		
Radiation	Exposure to ionizing radiation, such as from nuclear accidents or medical procedures, can lead to upregulation of several enzymes, including NOX and NOS, that generate ROS [99]. ROS is also induced by ETC complexes, and decreased SOD activity contributes to higher ROS levels [99,100].	Fibrosis significantly increases the risk of coronary artery disease, cardiomyopathy, valvulopathy, arrhythmias, and pericardial disease [101,102,103,104].
**Chemical Waste and Spills**		
Chemical Waste and Spills	Some organic solvents used in industrial processes, like benzene and toluene, can produce ROS when metabolized in the body, contributing to oxidative stress and potential toxicity. Chemical waste-induced ROS is often produced by NOX, CYP, ETC complexes, peroxisomal oxidases, and XO. ROS also increases due to reduced SOD and CAT activity [105,106,107,108].	Mice inhaling volatile benzene had significantly reduced levels of circulating angiogenic cells (Flk-1^+^/Sca-1^+^) and increased levels of plasma LDL [109].
**Microplastics**		
Microplastics	Microplastics induce ROS production via ETC complexes. ROS also increases due to reduced SOD, GPx, and CAT activity [110,111].	Cardiac fibrosis and damage in Wistar rats [112].
		Damaged cardiac structure and function [113].
		Cardiotoxicity, pericardial edema, and impaired heart rate in fish cardiac tissue [114].

## Abbreviations

The following abbreviations are used in this manuscript:

BPA	Bisphenol A
CAT	Catalase
CD	Cadmium
CHD	Coronary heart disease
CVD	Cardiovascular disease
EDCs	Endocrine-disrupting chemicals
ETC	Mitochondrial electron transport chain
GPx	Glutathione peroxidase
GSH	Glutathione
Gst	Glutathione S-transferase
NO	Nitric oxide
NOX	NADPH oxidase
NSAIDs	Non-steroidal anti-inflammatory drugs
PCBs	Polychlorinated biphenyls
PM	Particulate matter
PFCs	Perfluorinated compounds
PFOS	Perfluorinated compound perfluorooctane sulfonate
SOD	Superoxide dismutase
TrxR	Thioredoxin reductase
XO	Xanthine oxidase
